# Exploring the Role of Vitamin D in Familial Mediterranean Fever: Pathogenesis, Triggers, and Immune Modulation

**DOI:** 10.3390/medsci14020279

**Published:** 2026-05-31

**Authors:** Hagop Sassounian, Saad Aad, Hilda E. Ghadieh, Lara Khouzami, Elsa Nicolas, Sami Azar, Frederic Harb

**Affiliations:** 1 Department of Biomedical Sciences, Faculty of Medicine and Medical Sciences, University of Balamand, Tripoli P.O. Box 100, Lebanon; 2 Department of Biological and Physical Sciences, School of Arts and Sciences, American University in Dubai, Dubai P.O. Box 28282, United Arab Emirates; 3 Department of Health Sciences, College of Natural and Health Sciences, Zayed University, Dubai P.O. Box 144534, United Arab Emirates; 4 Department of Life and Earth Sciences, Faculty of Sciences, Lebanese University, Fanar, Jdeideh P.O. Box 90656, Lebanon

**Keywords:** Familial Mediterranean Fever, MEFV gene, vitamin D deficiency, epigenetic modifications, autoinflammatory diseases, immune modulation

## Abstract

Familial Mediterranean Fever (FMF) is among the most frequent autoinflammatory diseases in populations originating from the area of Middle Eastern and Mediterranean countries. It is caused by mutations in the MEFV gene, which causes dysregulated pyrin expression and thus an immunologic anomaly. FMF is diagnosed by recurrent episodes of fever and serosal inflammation, predominantly peritonitis and pleuritis, as well as other systemic symptoms. Recent research is dedicated to searching for factors beyond genetic code contributing to how FMF evolves, the severity of its symptoms and response to conventional therapy—colchicine. These factors include epigenetic modifications of the MEFV gene and other environmental factors, such as cold exposure, stress, composition of gut flora and diet. Among these factors, vitamin D, best known for its classical role in musculoskeletal health, has emerged as a powerful immune modulator. It has been documented that vitamin D has been implicated in the regulation of pro-inflammatory cytokines and may modulate immune responses. Notably, in regions with some of the highest reported prevalences of MEFV mutations—likely reflecting Mediterranean populations more broadly—vitamin D concentrations are frequently low. This overlap raises the hypothesis that vitamin D deficiency may be associated with FMF pathogenesis, although current data are largely correlational and do not establish causality. In this review, we summarize current evidence on FMF pathogenesis, potential triggers, and vitamin D metabolism, and explore how vitamin D may modulate immune responses and intersect with key autoinflammatory pathways, considering whether adequate vitamin D supplementation could help reduce disease burden in some patients with FMF.

## 1. Introduction

Familial Mediterranean Fever, known as FMF, is among the most frequent hereditary autoinflammatory diseases. It is a Mediterranean disease that primarily affects Turks, Jews, Armenians and Arabs. It presents with recurrent episodes of fever and serosal or articular inflammation, most commonly manifesting as peritonitis, pleuritis, or synovitis.

FMF primarily affects populations from the Mediterranean region, with Türkiye accounting for a substantial proportion of reported cases. The prevalence of FMF among Turkish population is about 1/1000 and the highest prevalence has been reported in Central Anatolia with a prevalence of 1/395 [1]. This was also confirmed in another study, where some areas of Turkey, being classified as Central Anatolia, reached a prevalence of FMF as high as 8.8/1000 [2]. The frequency of FMF among Armenian population is approximately 1/150, and the proportion of gene carriers for the disease could be even higher, reaching 1/3 [3]. Higher rates have been reported in certain populations, including Sephardic Jews in Israel (approximately 1 in 1000), while lower rates are observed among Ashkenazi Jews. There is scarce recent data about Arab Mediterranean countries, including Syria, Lebanon and Jordan, despite FMF being one of the most common periodic diseases in these countries [4].

FMF is caused by a gain-of-function mutation in the MEFV gene located on chromosome 16p. The MEFV gene consists of 10 exons, and mutations are most frequently found in exons 2 and 10. Several epidemiological reports have indicated that the M694V polymorphism of exon 10 in the MEFV gene mutation is the most common among populations in the Mediterranean basin. Other less frequently occurring mutations are E148Q in exon 2, M690I and V726A in exon 10 [5].

The protein encoded by the MEFV gene is known as pyrin. Pyrin is a granulocyte protein that acts by the inflammasome that causes pro-inflammatory cytokine production. Recent studies have shown that mutation in the MEFV gene leads to an overactive pyrin, thereby initiating enhanced immune responses due to exaggerated IL-1β and IL-18 production [5,6]. This is in contradiction to previously published studies, which suggested that impaired function of pyrin caused the pathogenesis of FMF, based on the hypothesis that pyrin physiologically counteracts production and activation of IL-1β, and mutated variants do not suppress the release of inflammatory mediators [6].

FMF is generally manifested by episodic attacks of fever and widespread inflammation throughout the body, whether it be peritonitis, pleuritis and/or arthritis. The disease often occurs in childhood, with symptoms emerging as an episode which can last from 12 h to 3 days and usually resolve spontaneously [5].

Fever is the most common symptom, as it is present in almost all patients during attacks. Abdominal pain is the second most common manifestation, which is due to the inflammation of the peritoneum, which elicits a diffuse abdominal discomfort. This diffuse pain can often mimic symptoms of an acute abdomen or appendicitis, with rebound tenderness, distention of the abdomen and decreased bowel sounds. In fact, it was found that the number of appendectomies performed in patients with FMF was higher than in the normal population, and the majority of the appendices removed in these patients were not inflamed in the first place [7].

Pleuritis and pericarditis are less frequent symptoms. They often present late in the disease course, manifesting with dyspnea and friction rub at the site of the pleuritic inflammation, in addition to retrosternal chest pain. ST changes can also be seen in electrocardiograms during attacks. Other symptoms include arthralgia/arthritis, myalgia, orchitis, and erysipelas-like erythema [5].

Colchicine has remained the first-line treatment for Familial Mediterranean Fever since 1972. It is known to disrupt microtubule assembly in neutrophils and to inhibit the pyrin inflammasome formation, thus preventing the production of pro-inflammatory cytokines such as IL-1β. Colchicine works primarily by preventing attacks and accumulation of serum amyloid A protein (SAA) in different tissues of the body, with AA amyloidosis being the most serious complication in patients with untreated FMF. In cases where patients are intolerant or resistant to colchicine, newer therapeutic methods have emerged. One category of drugs to treat colchicine-resistant cases is anti-IL1 medications. Anakinra, for example, is a recombinant, non-glycosylated homolog of the human IL-1 receptor antagonist. It works by blocking the IL-1Ra, which prevents the activation of the IL-1 signaling pathway, ultimately inhibiting pro-inflammatory responses. Other anti-IL1 drugs include Rilonacept and Canakinumab, a human IL-1β receptor fusion protein and a human monoclonal anti-IL-1β antibody respectively [2].

Although FMF has been extensively studied from its genetic aspect, there remains a significant gap in research addressing the epigenetic factors that may contribute to its pathogenesis. The recent literature is turning its focus to how certain epigenetic factors influence disease severity and progression. This becomes even more relevant when environmental factors come into play as well. In this context, this article focuses on the epigenetic regulation in FMF, emphasizing DNA modifications such as methylation and histone modification. The review also addresses the importance of environmental factors, shedding light on one important hormone, which is vitamin D, aiming to understand how it is involved in immune pathways and how its deficiency contributes to FMF pathogenesis.

## 2. Triggering Factors

FMF is known to be inherited in an autosomal recessive fashion, where one individual needs to have two mutated copies of the MEFV gene in order to manifest the disease. However, several cases have been reported where people develop the disease having only one mutated variant of the MEFV gene [8]. It was also found that different genotypic variants of the MEFV gene resulted in distinct disease presentations, with certain mutations presenting with more severe symptoms, more frequent attacks and more susceptibility to conventional treatment failure [9]. Although genetic variability plays a significant role in the phenotypic differences of FMF, several triggering factors other than the genetic code itself have also been identified and their roles have been studied in the pathogenesis of FMF.

### 2.1. Epigenetics

Epigenetic modifications are changes outside of the DNA sequence that alter gene activity. They act like molecular switches that can turn genes on or off depending on environmental, developmental, or cellular signals [10]. Here, we highlight two important epigenetic factors that help us explain why individuals with the same genetic mutation can still show different disease severity, symptoms, or treatment responses.

*1.*  *DNA Methylation*

The first epigenetic modification to be discussed is DNA methylation. The exact contribution of methylation/demethylation to Familial Mediterranean Fever presentation is controversial until this day, with several opposing hypotheses mentioned in the literature. Researchers have suggested that the NLRP3 inflammasome, which will be discussed extensively in coming parts, is expressed in higher amounts when the gene responsible for its production is demethylated, leading to more severe symptoms [11].

Caldiran F. et al. (2024) have studied another entity called G protein regulator signal 10 (RGS10) and its role as an immune regulator in FMF [12]. RGS10 is known to have anti-inflammatory properties, and it was found that it was present in larger quantities in patients with FMF compared to healthy individuals. Studying the gene coding for RGS10 revealed that controls had higher methylation levels compared to patients with FMF. This can be explained as a type of defense mechanism of the body, upregulating the expression of RGS10 to reduce the inflammatory process in FMF by demethylating the gene responsible for its production [12].

Another study done in Egypt has shown that methylation of the MEFV gene was correlated with more severe disease course, non-response to colchicine and higher levels of inflammatory markers such as CRP, ESR and serum amyloid A (SAA). However, these results were statistically non-significant, presumably due to their small sample size, warranting further research about the exact mechanism of how DNA methylation plays a role in the pathogenesis of FMF [11,13].

*2.*  *Histone Modification*

Histone-level epigenetic regulation is another important factor that appears to play a significant role in shaping inflammatory responses in Familial Mediterranean Fever (FMF). Findings from Caldiran et al. (2024) provided direct experimental evidence for dysregulated histones in patients with FMF, particularly during inflammatory attacks [12]. The authors showed that in patients with FMF, peripheral blood mononuclear cells have high levels of pro-inflammatory histone modifications—particularly H3K4me3 and H3K14ac, known to be enriched at transcriptionally active chromatin [12]. As shown by Caldiran et al., the upregulation of these stimulatory alterations may enable increased transcription of inflammatory genes and thus lower the threshold for cytokine elaboration, which can in turn lead to exaggerated clinical features on FMF attacks seen in certain patients.

These observations are consistent with a more general perspective as described in Chaaban et al. (2024), who also reviewed emerging evidence for FMF being under complex epigenetic regulation that goes beyond DNA methylation [11]. Their review emphasizes that histone modifications contribute together with other epigenetic host factors—such as chromatin accessibility changes—to an increased activation of the innate immune system. Chaaban et al. specifically emphasized that increases in permissive histone marks such as H3K4me3, observed in several autoinflammatory conditions, may allow rapid transcription of inflammasome-related genes, making monocytes and neutrophils more reactive to inflammatory stimuli [14].

### 2.2. Environmental Factors

Despite the fact that Familial Mediterranean Fever is a combination of different genetic and epigenetic modifications, research suggested that there are certain external influences as well that can act as triggering factors, altering disease presentation and affecting treatment response [15,16].

In a study conducted by Farisogullari et al. (2023), it has been shown that 78% of the patients with FMF included in their study had at least one triggering factor [17]. These observations were further reinforced by another study published by Parlar et al., having a larger cohort for the study, which consisted of 882 adults with FMF [18]. Their results showed that around 85% of participants complained of two or more factors precipitating their attacks. Furthermore, patients who did not respond to colchicine reported having more triggering factors than colchicine-sensitive patients with FMF [18].

In this section, we review some of the environmental determinants that have been frequently implicated in the pathogenesis of FMF across published studies.

*1.*  *Stress.*

On a psychological level, emotional stress is recognized as one of the significant triggers, as it activates the sympathetic nervous system, leading to catecholamine release such as epinephrine and norepinephrine, ultimately stimulating inflammatory pathways that exacerbate FMF symptoms. Cumulative clinical evidence suggests that nearly two-thirds of patients with FMF experience attacks precipitated by emotional stress; in some colchicine-unresponsive patients with comorbid depression, selective serotonin reuptake inhibitors (SSRIs) have been used to reduce attack frequency [15].

*2.*  *Exposure to Cold Temperatures*

Another triggering factor, which is also indirectly linked to sympathetic nervous system activation, is cold exposure. It has been reported as the most common trigger in several studies. A significant observation in one study was that almost half of the patients who reported cold exposure as a triggering factor were colchicine-resistant in terms of treatment and benefited greatly from more novel medications such as IL-1 antagonists [17].

*3.*  *Diet*

Eating habits are also frequently discussed in the literature. Studies proposed that patients report more frequent and severe attacks when they consumed diets rich in saturated fats, processed meats and refined sugars. This high consumption results in inflammation and oxidative stress, which are key contributors to the development and exacerbation of FMF symptoms [19]. Other studies have also shown that inflammatory responses are significantly mediated by high-salt intake, and they worsen the severity of the disease [20]. Interestingly, one study found significantly higher BMI and central adiposity in patients with FMF than in healthy controls [19]. Moreover, the same study demonstrated that chronic inflammation in FMF is associated with the continuous production of pro-inflammatory cytokines, mainly interleukin-1 beta (IL-1β) and Tumor Necrosis Factor alpha (TNF-α), which are always being produced. These cytokines can influence genes involved in lipid metabolism, promoting increased fat accumulation. The persistent inflammatory state may contribute to the development of insulin resistance, thereby exacerbating metabolic disturbances in affected individuals [19]. Another interesting study suggested an association between gut microbiota composition, disease phenotype, and treatment response in patients with FMF. Individuals with FMF showed a higher abundance of several pro-inflammatory bacteria compared with controls, particularly within Proteobacteria phylum and the Enterobacteriaceae family, including Enterobacter and Klebsiella. These findings suggest that gut microbiota alterations may contribute to FMF inflammation and influence treatment outcomes in patients [21].

In contrast, diets that are rich in fresh fruits, leafy vegetables and healthy fats have been proven to alleviate FMF symptoms and lead to better well-being of patients [22]. This is primarily due to the anti-inflammatory and antioxidant factors present in those nutrients. Some food supplements have also shown some benefit among people with less severe symptoms and fewer attacks [19].

For example, vitamin D (or calcitriol or 1,25-dihydroxycholecalciferol) is a cholesterol-derived fat-soluble molecule with multiple essential roles in the human physiology. Its functionality ranges from calcium and phosphate metabolism to bone formation, cell growth arrest, and cellular differentiation. Current research is exploring the roles of vitamin D beyond the musculoskeletal system, as focus is being shifted towards how vitamin D influences the immune system. Vitamin D has been implicated in the regulation of immune responses, including the modulation of pro-inflammatory cytokines and the differentiation of immune cells. Moreover, it was found to have a protective effect by attenuating the generation of reactive oxygen species (ROS), thereby limiting apoptosis and inflammatory processes [23].

In a related context, it has been historically known that the Middle East and Northern Africa (MENA) region has the highest prevalence of vitamin D deficiency. Coincidentally, these same geographical areas also report the most cases of patients with FMF. Therefore, this review aims to explore the possible pathways that might be common and overlapping between the sites of action of vitamin D in immune modulation and inflammation and the pathogenesis of FMF.

## 3. Vitamin D and Familial Mediterranean Fever

### 3.1. Mechanistic Overview of Vitamin D and Its Immunomodulatory Functions

Vitamin D is often considered more of a hormone than a vitamin, as it is produced in the skin through exposure to UVB light, and, in smaller proportion, through dietary sources in the form of vitamin *D*_2_ and vitamin *D*_3_. The first product of vitamin D metabolism is formed in the liver, 25-hydroxyvitamin D, which is the main circulating form of vitamin D. In the kidney as well as in other extra-renal tissues such as immune cells, it is further converted into its biologically active form, 1,25-dihydroxyvitamin D. This active form is clinically measured as a reference marker for assessing vitamin D concentrations. However, Vitamin D concentrations show considerable variability between individuals as they are influenced by multiple factors, including lifestyle and genetic backgrounds. Indeed, some studies have suggested the presence of genetic variants involved in vitamin D metabolism and its transport amongst adults [24].

Characteristically, vitamin D stabilizes calcium, phosphate, and bone health by increasing intestinal calcium absorption, which supports mineralization, and prevents secondary hyperparathyroidism. Several other studies also claim that vitamin D holds important extra-skeletal roles, particularly in the immune systems of children with pediatric rheumatologic conditions [24].

Monocytes, macrophages, dendritic cells, and many other immune cells express the vitamin D receptor (VDR) and the enzyme CYP27B1. This allows these cells to locally convert 25-hydroxyvitamin D to its active form, 1,25-dihydroxyvitamin D, enabling them to respond in both autocrine and paracrine ways [24]. Vitamin D influences immune function by binding to the VDR and modifying gene expression, which is able to program immune cell gene expression through VDR-mediated changes in gene expression and through epigenetic mechanisms. As a result, vitamin D signaling can regulate hundreds of genes involved in both innate and adaptive immune responses.

Functionally, this translates into a broad immunomodulatory profile that is summarized in Table 1:

Overall, Vitamin D’s ability to enhance antimicrobial defense while dampening excessive inflammation explains why many studies consider it not only a nutrient and a hormone but also a powerful immunomodulator, and why it is regarded as a potent modifier of autoimmune and autoinflammatory diseases [24,25].

Large-scale clinical data are consistent with a role in immune-mediated disease: in the VITAL trial, daily vitamin *D*_3_ (2000 IU) versus placebo in generally healthy adults led to a modest but significant reduction in incident autoimmune diseases over a median of 5.3-year follow-up [26]. Although it may not be considered as definitive or irrefutable “proof” across all conceivable diseases and conditions, these findings clearly support the idea that preserving an optimal level of vitamin D has the potential to substantially help in regulating and controlling immune activation.

### 3.2. Vitamin D Deficiency

The biochemistry of vitamin D deficiency is characterized by a low serum level of 25-hydroxyvitamin D. Different cut-offs are used, but many authors consider levels below 20 ng/mL as indicative of deficiency or states that can be corrected with supplementation, while levels between 20 and 29 ng/mL are classified as insufficiency. Broad reviews confirm that this deficiency is a global phenomenon, even in sun-rich regions, with implications for bone health, cardio-metabolic function and immune outcomes [27,28].

Skeletal manifestations across age groups are summarized in Table 2:

Other unspecific manifestations have also been noted in the majority of patients, and these include fatigue, diffuse musculoskeletal pains, myalgias, proximal muscle weakness, mood disturbances and increased incidence of respiratory tract infections [28,30]. Moreover, chronic musculoskeletal pain, sarcopenia, and poor function in patients with inflammatory and autoimmune rheumatic diseases have all been associated with low vitamin D concentrations [31].

Vitamin D deficiency has also been associated (with varying levels of evidence) with an increased risk or severity of several immune-related conditions, including respiratory infections, inflammatory bowel disease, multiple sclerosis, rheumatoid arthritis and other immune disorders [24,25]. The VITAL trial suggests that long-term Vitamin D supplementation may slightly reduce the incidence of new autoimmune diseases, suggesting that chronically low vitamin D status is not neutral for immune function [26].

Vitamin D deficiency is especially prevalent in groups with limited sun exposure (indoor lifestyle, conservative clothing), darker skin pigmentation, obesity, older age, and chronic illnesses, patterns that have been consistently documented in epidemiologic and mechanistic studies [28,30].

Lebanon is a good example of this “sunny but deficient” paradox. A large study from Greater Beirut adults between 2014 and 2016 found that the prevalence of vitamin D deficiency was very high, despite abundant sunlight, and was particularly marked in women and older individuals [32]. More recent data from a tertiary center spanning the pre- and post-COVID-19 periods confirm that a large proportion of Lebanese adults still have suboptimal 25 (OH)D levels; although there was some improvement over time, deficiency and insufficiency remain common [33].

Genetic studies in older Lebanese men and women further show that multiple exonic variants in vitamin D pathway genes contribute to inter-individual variability in 25 (OH)D levels [34], reinforcing the idea that some populations may be biologically predisposed to vitamin D deficiency when combined with environmental conditions such as indoor living, air pollution, and reduced sunlight exposure during winter.

### 3.3. Vitamin D Status in FMF and Overlap with Deficient Populations

Familial Mediterranean Fever (FMF) is most prevalent in populations around the eastern Mediterranean basin, as described earlier. These populations share exactly the same environmental and lifestyle risk factors for vitamin D deficiency: high rates of indoor work or study, conservative clothing, limited midday sun exposure, and, in some groups, higher rates of obesity and metabolic syndrome [28,32].

Importantly and as previously mentioned, FMF is an autoinflammatory disorder characterized by dysregulated inflammasome activation and IL-1-dependent inflammation. Vitamin D insufficiency is a common finding in children with chronic rheumatic and autoinflammatory diseases and may be involved in the enhancement of inflammatory response leading to musculoskeletal pain [24]. Consequently, this hypothesis might be in accordance with more general studies reporting that low levels of vitamin D can be especially high in these patients [25,31]. These findings support a biologically plausible modulatory role of vitamin D on inflammatory pathways but do not, on their own, establish a causal effect on FMF activity.

In this regard, a study conducted among pediatric population with FMF reported that the mean serum levels of 25-hydroxyvitamin D were significantly lower in children with FMF compared with healthy controls, with a large proportion of these patients falling within the deficient range (<20 ng/mL) [35]. While causality cannot be established from this finding, these cross-sectional data suggest that children with FMF may represent a group in whom vitamin D deficiency is common, but they do not clarify the direction of association or exclude confounding factors.

Layering this together, we summarized data from the literature in Table 3:

Henceforth, vitamin D-deficient populations and FMF-affected populations overlap both geographically and clinically: Mediterranean people, who frequently suffer from vitamin D deficiency and are also affected by FMF, suffer more commonly from recurrent painful episodes driven by cytokine activity. In such scenario, an adequate level of vitamin D is not limited to bone, but is also a plausible, low-risk way to help with immune system homeostasis [38].

### 3.4. Common Immune Pathways Between FMF Pathogenesis and Vitamin D Regulation

In FMF, the inflammatory phenotype arises from the convergence of several reinforcing innate and adaptive immune pathways. The process begins with mutations in the MEFV gene, which encodes pyrin, a regulatory protein involved in inflammasome control. Pathogenic variants impair pyrin’s inhibitory regulation, lowering the activation threshold of the pyrin inflammasome. This results in enhanced caspase-1 activation and increased cleavage of pro-IL-1β into its active form, thereby sustaining an IL-1β-driven inflammatory state. Persistent IL-1β signaling promotes hepatic acute-phase reactant production and explains why markers such as CRP, SAA, and PTX3 may remain elevated even during clinically quiescent periods [36].

Beyond innate immune activation, chronic IL-1β exposure reshapes adaptive immunity. IL-1β, together with IL-6, favors differentiation of naïve CD4^+^ T cells toward a Th17 phenotype. This polarization is particularly evident in patients with severe genotypes such as M694V homozygosity, who demonstrate enhanced IL-17 production and amplified neutrophilic responses [39]. IL-17 further recruits and activates neutrophils, reinforcing serosal inflammation and perpetuating the inflammatory loop.

Thus, FMF is characterized not only by episodic inflammasome activation but also by a persistently primed immune milieu in which innate IL-1β signaling and adaptive Th17 polarization synergistically sustain inflammation [39].

**Figure 1:**
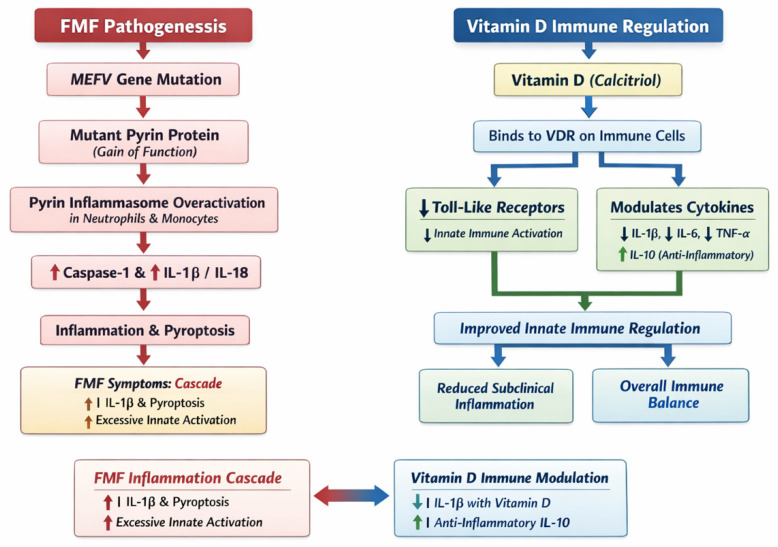
Proposed mechanism of molecular changes in FMF pathogenesis and the influence of Vitamin D as an immune modulator in regulating inflammatory responses.

There is also a more complicated interaction between pyrin and NLRP3, with some reports suggesting that FMF leukocytes alter NLRP3 expression. Moreover, NLRP3-mediated IL-1β release can actually be reduced, as an attempt of the immune system to compensate for the hyperactive pyrin pathway [40]. When combined with the persistent oxidative stress documented in FMF [36], the net effect is an immune environment heavily skewed toward IL-1 and IL-6 signaling, enhanced Th17 polarization, and neutrophil-driven inflammation. In this context, the inflammasome network is primed and more readily activated compared to physiological conditions. These interactions are summarized schematically in Figure 1.

Vitamin D physiologically modulates several inflammatory pathways. In patients with FMF and related conditions, small experimental and clinical studies suggest that vitamin D supplementation can be associated with reductions in IL-1, IL-6, and TNF-α and increases in IL-10 expression [37]. Immune cells can locally convert vitamin D into its active form during inflammation, suggesting that vitamin D functions as an intrinsic regulatory mechanism to limit excessive cytokine release, which influences T-cell differentiation and Toll-like receptor (TLR) signaling, both of which operate upstream of key innate immune responses [40].

In the context of vitamin D insufficiency—consistently reported in both adult and pediatric FMF cohorts [37]—these regulatory mechanisms become less effective. In a pediatric study by Mohamed et al. (2020), lower levels of vitamin D were associated with higher PTX3 concentrations, and increased oxidative stress (ROS) correlated with both elevated PTX3 and reduced vitamin D concentrations in children [37]. These findings support the idea that an immune system already primed by a dysregulated pyrin signaling and increased IL-1 activity may be more prone to exhibit an exaggerated immune response when vitamin D is insufficient, as low vitamin D concentrations may impair the suppression of IL-1, IL-6 and reduce the induction of Il-10, thereby facilitating the expansion of preexisting pro-inflammatory T-cell subsets, including Th17 cells [39].

Oxidative and inflammasome pathways add another layer. FMF cells generate more ROS, which can activate or amplify inflammasome signaling [36]. The pediatric FMF data showed that oxidant status dropped once vitamin D rose above about 20 ng/mL, which implies that vitamin D helps buffer oxidative stress in FMF [37]. Because mitochondrial damage and ROS influence inflammasome behavior, improving vitamin D concentrations may indirectly stabilize inflammasome activity in pyrin-mutant cells. Vitamin D also modulates TLR responses. In states of deficiency, TLR-driven cytokine production becomes exaggerated, which further amplify the IL-1 loop [40].

Additional aspects of FMF physiopathology are linked to vitamin D signaling. Vitamin D binding protein (VDBP), which carries most circulating vitamin D metabolites, also influences neutrophil trafficking, macrophage activation, and lymphocyte function. In patients with FMF lacking *MEFV* mutations, one VDBP allele (allele 2) appears more frequently and has been associated with amyloidosis and arthritis [41]. In people with vitamin D deficiency, such genetic variation may further reduce the effective delivery of active vitamin D to immune cells, allowing greater neutrophil-mediated inflammation.

Regulatory miRNAs play a vital role as well. Patients with FMF present altered plasma levels of miR-155 and miR-204 in [42,43]. Studies have shown that miR-204-3p suppresses LPS-induced cytokine production through PIKγ in FMF. While data linking vitamin D to miRNA regulation is limited, vitamin D is known to modulate miRNA expression in other inflammatory settings, meaning that a deficiency could potentially shift these networks toward more cytokine output.

Available data suggests that vitamin D supplementation may be beneficial in FMF, but results are still preliminary and need to be validated in larger clinical trials In randomized studies of patients with attack-free FMF who adopted an anti-inflammatory diet in combination with vitamin D supplementation, serum vitamin D levels increased, CRP levels decreased and attack frequency, duration and overall severity improved, along with enhanced cognitive performance scores [22]. Even if diet itself had multiple components, the equivalent improvement in vitamin D and inflammatory markers aligns with the overall model: FMF involves pyrin-inflammasome signaling, IL-1/Th17 pathways, neutrophil-dominated inflammation, and oxidative stress; vitamin D deficiency removes key regulatory checkpoints across these pathways. Thus, maintaining adequate vitamin D concentrations in patients with FMF has a strong biological rationale, not only for skeletal health, but also for reducing background inflammation, limiting oxidative stress, and even lowering the long-term risks such as amyloidosis and vascular complications.

## 4. Limitations

Several limitations of this review should be acknowledged. First, the available clinical evidence on vitamin D in FMF is predominantly derived from small, cross-sectional, and observational studies with limited sample sizes, making it difficult to draw firm conclusions about directionality or causality, as observational designs are inherently susceptible to confounding and reverse causation [27,28]. Second, the absence of adequately powered randomized controlled trials specifically addressing vitamin D supplementation in FMF represents a major evidence gap; as noted in the broader vitamin D literature, RCTs in this area are difficult to conduct and often yield inconclusive results due to heterogeneous baseline vitamin D status, variable dosing regimens, and differences in supplementation duration and adherence [44,45]. Third, important confounding factors—including BMI, dietary habits, sun exposure, skin pigmentation, clothing practices, and co-existing metabolic conditions—are rarely controlled for in FMF-specific vitamin D studies and are known to independently influence circulating 25 (OH)D levels [28,32]. Fourth, population heterogeneity across FMF cohorts, in terms of genotype severity, disease activity, colchicine adherence, and ethnic background, limits the generalizability of findings. Finally, lack of standardization in vitamin D measurement methods—including variability between immunoassay-based platforms and liquid chromatography–mass spectrometry approaches—introduces inter-laboratory variability that complicates cross-study comparisons and may lead to misclassification of vitamin D status [46,47]. These limitations collectively underscore the need for prospective, well-designed, and adequately powered trials before any clinical recommendations regarding vitamin D monitoring or supplementation can be established in patients with FMF.

## 5. Conclusions

Although FMF is considered a strict genetic autoinflammatory disease, other biological and environmental elements may contribute to its etiopathogenesis when combined. Such determinants and other modifying agents and their targeted management may be expected to decrease the frequency and severity of specific FMF phenotypes. Vitamin D, an important hormone with immunomodulatory consequences, is a plausible candidate for modulating inflammation in FMF. Elucidating its exact mechanisms of action and defining optimal supplementation strategies may reveal potential therapeutic benefits, but at present, the clinical evidence is limited and largely observational. Future adequately powered randomized and longitudinal studies are needed to determine whether improving vitamin D status can causally influence FMF activity and long-term outcomes. Further studies are needed to identify how vitamin D status directly influences disease activity and quality of life in FMF.

## Figures and Tables

**Table 1:** Immunomodulatory effects of vitamin D on innate and adaptive immune responses.

Immune System Component	Effect of Vitamin D	Mechanism/Key Actions	Representative References
Innate immunity—monocytes, macrophages, neutrophils	Enhances antimicrobial defense; attenuates excessive inflammatory responses	Upregulates antimicrobial peptides (cathelicidin, β-defensins); modulates Toll-like receptor signaling; reduces excessive production of pro-inflammatory cytokines (IL-1β, IL-6, TNF-α); promotes phagocytosis	[24]
Dendritic cells	Promotes a more tolerogenic phenotype	Reduces expression of MHC class II and co-stimulatory molecules; decreases IL-12 production; favors induction of regulatory T cells over Th1/Th17 priming	[24]
NK cells	Modulates cytotoxic and cytokine-secreting functions	Regulates expression of activating/inhibitory receptors; may reduce excessive IFN-γ production while preserving antimicrobial activity	[25]
Adaptive immunity—Th1 cells	Down-regulates Th1-polarized responses	Decreases IL-2 and IFN-γ production; shifts CD4^+^ T-cell profile away from Th1-dominant inflammation	[24]
Adaptive immunity—Th17 cells	Attenuates Th17 responses	Reduces IL-17 production and differentiation of Th17 cells, thereby limiting neutrophil-driven inflammation and tissue damage	[24,25]
Regulatory T cells (Tregs)	Enhances regulatory and anti-inflammatory circuits	Promotes expansion and function of FoxP3^+^ Tregs; increases production of IL-10 with downstream suppression of pro-inflammatory cytokines	[24]
B cells	Modulates antibody production and antigen presentation	Inhibits plasma-cell differentiation and immunoglobulin production in some contexts; alters B-cell cytokine secretion and antigen-presenting capacity	[25]

**Table 2:** Selected clinical consequences of severe vitamin D deficiency across age groups.

Population/Age Group	Major Clinical Consequences	Typical Manifestations	Key References
Infants and children	Rickets; impaired skeletal development	Bone deformities (genu varum/valgum), delayed motor milestones, growth retardation, widened wrists/ankles on exam or radiography	[29]
Adolescents	Subclinical osteomalacia; reduced peak bone mass	Diffuse bone pain, fatigue, increased fracture risk with minor trauma; reduced bone mineral density	[30]
Adults	Osteomalacia and increased fragility fractures	Proximal muscle weakness, difficulty climbing stairs or rising from a chair, low bone mineral density, atraumatic fractures	[30]
Older adults	Sarcopenia; falls; functional decline	Muscle weakness, impaired balance, higher risk of falls and hip fractures, loss of independence	[28]
Patients with inflammatory/rheumatic diseases	Worsening musculoskeletal pain and poor function	Chronic widespread pain, fatigue, lower physical performance, overlapping with inflammatory musculoskeletal complaints	[31]
Immune-related conditions (general)	Increased susceptibility to infections and immune-mediated diseases (associations)	Higher risk or severity reported for respiratory infections and various autoimmune diseases in observational studies	[24,26]

**Table 3:** Multilevel links between vitamin D deficiency and FMF in Eastern Mediterranean populations.

Level	Description/Relevance	Illustrative Evidence in FMF-Related Populations	References
Population level	High baseline prevalence of vitamin D deficiency and insufficiency in Eastern Mediterranean and MENA countries, including Lebanon and neighboring regions	Large Lebanese cohorts show high rates of suboptimal 25(OH)D concentrations despite abundant sunlight, especially in women and older adults	[32,33]
Environmental & lifestyle level	Shared risk factors for low vitamin D status overlap with FMF-endemic populations	Indoor work/study, conservative clothing, limited midday sun, air pollution, obesity and metabolic syndrome are frequent in the same ethnic groups where FMF is prevalent	[28,34]
Genetic level	Genetic variants influence both FMF and vitamin D pathways	MEFV mutations (e.g., M694V) drive pyrin-inflammasome activation, while polymorphisms in vitamin D-pathway genes modulate 25(OH)D concentrations and tissue responsiveness	[5,34]
Disease level (FMF cohorts)	FMF patients often show lower vitamin D concentrations than controls	Pediatric and adult FMF cohorts report significantly lower 25(OH)D concentrations, with a high proportion of patients in the deficient range (<20 ng/mL)	[24,35]
Pathophysiological level	Overlap between vitamin D-sensitive immune pathways and FMF mechanisms	Both FMF and vitamin D deficiency are linked to enhanced IL-1/IL-6 signaling, Th17 skewing, neutrophil-driven inflammation, and increased oxidative stress	[25,36,37]
Clinical/hypothesis level	Rationale for considering vitamin D as a low-risk modulator in FMF	Preliminary interventional data suggest that correcting vitamin D deficiency, often within broader anti-inflammatory interventions, may improve inflammatory markers and symptom burden in some FMF patients	[22,38]

## Data Availability

No new data were created or analyzed in this study.
